# Calculation method for novel upright CT scanner isodose curves

**DOI:** 10.1002/acm2.14377

**Published:** 2024-05-02

**Authors:** Michael W. Kissick, Costanza Panaino, Anthony Criscuolo, John Hayes, Carson Hoffman, Rockwell Mackie, Andries N. Schreuder

**Affiliations:** ^1^ Leo Cancer Care, Ltd. Middleton Wisconsin USA; ^2^ Department of Medical Physics University of Wisconsin – Madison Madison Wisconsin USA; ^3^ Engineering and Physical Sciences University of Surrey Guildford UK; ^4^ Medical Radiation Physics National Physical Laboratory Teddington UK

**Keywords:** CT isodose, CT upright tilting gantry, TOPAS Monte‐Carlo modeling

## Abstract

**Purpose:**

A computational method based on Monte‐Carlo calculations is presented and used to calculate isodose curves for a new upright and tilting CT scanner useful for radiation protection purposes.

**Methods:**

The TOPAS code platform with imported CAD files for key components was used to construct a calculation space for the scanner. A sphere of water acts as the patient would by creating scatter out of the bore. Maximum intensity dose maps are calculated for various possible tilt angles to make sure radiation protection for site planning uses the maximum possible dose everywhere.

**Results:**

The resulting maximum intensity isodose lines are more rounded than ones for just a single tilt angle and so closer to isotropic. These maximum intensity curves are closer to the isotropic assumption used in CTDI or DLP based methods of site planning and radiation protection. The isodose lines are similar to those of a standard CT scanner, just tilted upwards. There is more metal above the beam that lessens the dose above versus below isocenter.

**Conclusion:**

Aside from the orientation, this upright scanner is very similar to a typical CT scanner, and nothing different for shielding needs to be done for this new upright tilting CT scanner, because an isotropic scatter source is often assumed for any CT scanner.

## 1 INTRODUCTION

The purpose of this paper is to report on a computational method for producing the appropriate isodose curves for a new tilting upright CT scanner. These isodose curves enable radiation protection calculations and are usually the manufacturer's responsibility to provide. The isodose curves are air kerma curves since most x‐ray survey meters are calibrated for exposure and dose to air. The curves are dominated by scatter from a body sized water or tissue phantom at isocenter, and the primary beam is mostly stopped in the gantry itself. Therefore, it is scatter air isodose curves that are being calculated here.

The new scanner studied here is the Mary^®^ scanner from Leo Cancer Care, Inc. It can tilt by 15 degrees in addition to a basic upright (vertical) orientation. That orientation is very useful for radiation therapy treatment planning for upright treatments. Upright radiotherapy is gaining traction in proton and carbon ion therapy due to its cost saving potential (relative to rotating gantries).^1^, ^2^ It has also been indicated that upright radiotherapy may be better for patient comfort,^3^, ^4^ breathing control,^5^ and anytime when gravity acting along the body's length improves the anatomy or physiology.^6^ Consequently, there is interest in applying upright body positioning within photon radiotherapy also.^7^ Because the scanner can tilt for different patients, a maximum intensity dose for all tilt angles is proposed here to provide a single useful set of isodose curves.

The goal of this study is to update the isodose calculation method by using modern computation tools. The Monte‐Carlo method here uses a well‐established Monte‐Carlo code that recently comes with a useful wrapper to make its use easier. Unlike some other well‐established codes, there is no export control on the code used for this study, and that is important for an international company like Leo Cancer Care. More effort than this may not be required however.

Radiation protection is often based on CTDI (CT Dose Index) or DLP (Dose‐Length Product) based calculations that implicitly assume an isotropic scatter dose.^8^ Therefore, no matter how much the gantry tilts, those CTDI or DLP‐based calculations would not change at all. Only the isodose lines would change, and most sites will likely not modulate the wall shielding for the presence of the gantry attenuation anyway, because it could add restrictions to the future use of the space. Most sites will use the same thickness of lead or concrete in the walls for ease and flexibility. Due to this, the model could be simplified without compromising its accuracy.

## 2 METHODS AND MATERIALS

### 2.1 Mary scanner description

Leo Cancer Care, Inc., has designed a new upright CT scanner that can also tilt, called Mary^®^. The scanner has a bore of 85 cm and a field of view of 62.4 cm. The gantry can travel 180 cm, but this study considers just a single position in the middle of this range. The x‐ray tube can deliver a beam from 80 to 140 keV, but this study will only consider the highest energy of 140 kVp. The initial release will have a ±15 degree maximum tilt. The field width of the beam at isocenter is 19 mm wide.

**FIGURE 1:**
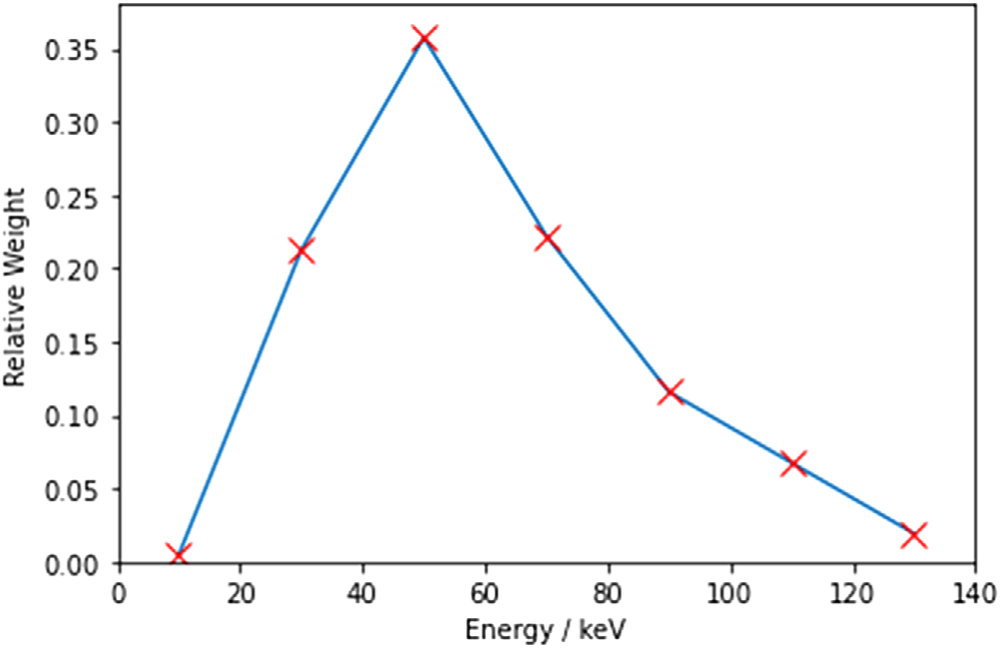
The approximate 140 kVp with 4 mm Al filter source spectrum used. The spectrum uses the standard available tool TASMICS with SPEKTR to get the seven discrete values shown with X marks. By accident, the upper tail bit between 130  and 140 keV is missing. The spectrum is loaded into TOPAS as “continuous,” putting linear interpolations between them.

The source spectrum used is shown in Figure 1. It approximates a 140 kVp spectra. The source spectrum used was originally calculated from TASMICS^9^ with the use of SPEKTR.^10^ The spectrum in the TOPAS code used these weights and specified a continuous and linear spectrum between them.

### 2.2 Monte‐Carlo calculations

The results in this paper are for a midpoint in its translation: 1.18 m off the ground. Only the minimum filtration was used for the simulations, just 4 mm of aluminum. The bowtie filter is included now but not added into the simulation for example. There are also lots of smaller, in terms of radiological density, components that were not added. Therefore, the numerical results of the simulation are considered preliminary coming into the study.

The TOPAS^11^ wrapper to Geant4 was used for these Monte‐Carlo calculations. The TOPAS code was chosen because it is based on the well‐validated code GEANT4.^12^ TOPAS wraps GEANT4 in an easier environment for those who prefer not to program in C++. It is considerably easier to use than other options. The code MCNP^13^ was considered, but the United States Department of Energy has a strict export control on the code making it a challenge for an international company that may use external technical support. The EGSnrc^14^ code and others that are also well‐validated were not considered because of the inability of those codes to import CAD files from the engineering designs. The main benefit of TOPAS is its ability to import STL type files of the actual engineering CAD files.

**FIGURE 2:**
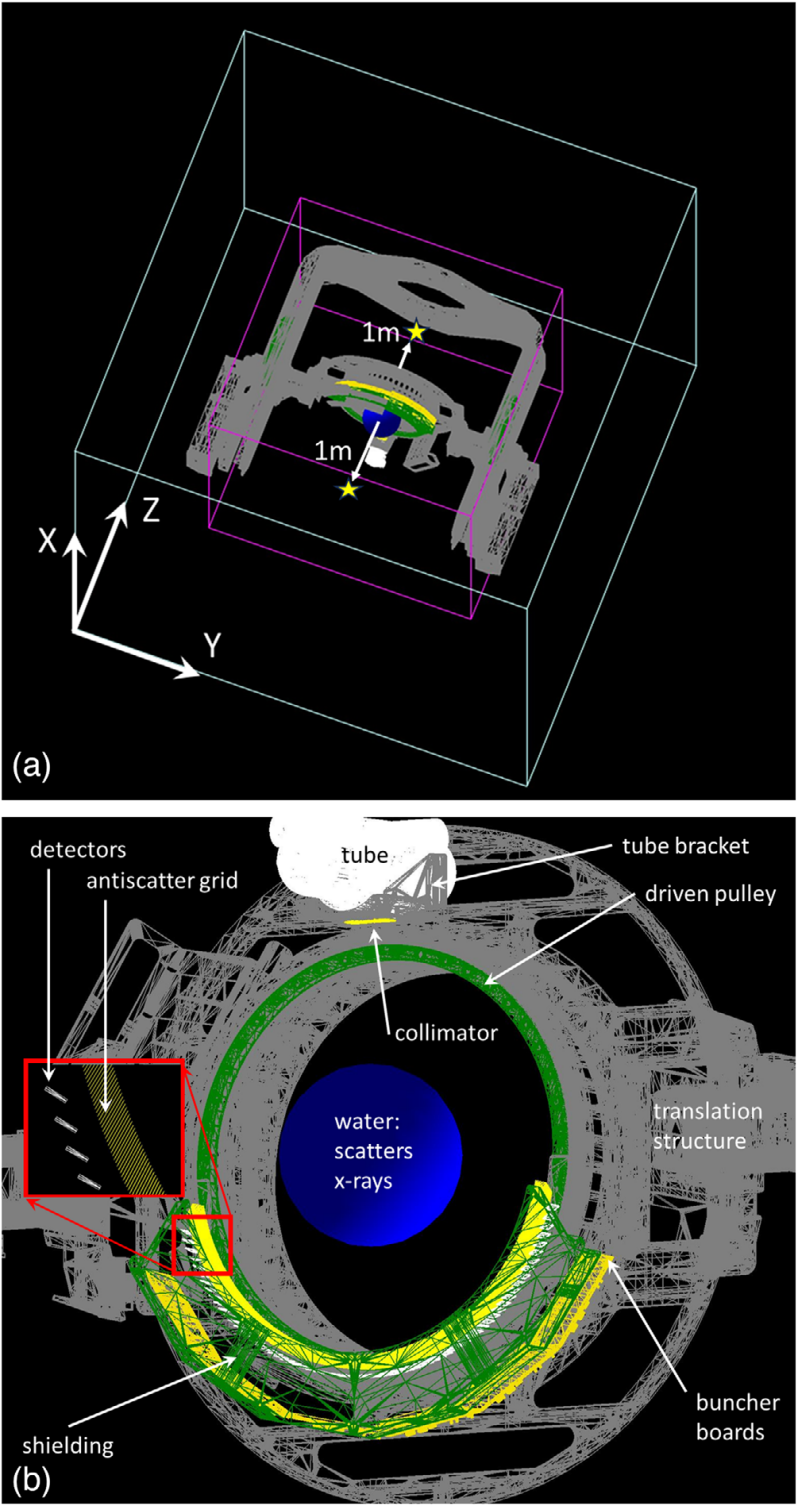
Using the native viewer in TOPAS, the calculation space (larger gray box in “a”) is shown with the entire calculation structure set listed in Table 1 in “a.” Also shown in “a” is the dose scoring space outlined in magenta and the locations of the measurements, the lower one is used for normalization. Note that the scoring space is a so‐called “parallel world” in the code that only contains air just for dose scoring. The sub‐figure “b” is a zoomed‐in view of just the gantry components from underneath. The gantry position shown is at 0 degrees tilt orientation. Note the axes direction in “a” indicated. Also indicated in “a” are the locations of the 1 meter out of the bore measurements.

The scoring region is handled as a “parallel world” in TOPAS. This parallel world is a cube of air centered at the center of the calculation space, ±1.45 m by ±1.45 m and ±1.35 m high in Z. The resolution of the dose scoring is 1 cm in each of the three directions. The whole radiation transport calculation space is an air cube with sides 5 m long, with 1 mm voxel resolution. The dose scoring parallel world is a smaller volume inside the calculation space. The rendering of the calculation space, the dose scoring space, and the other objects as well as the measurement locations is shown in Figure 2.

The calculations were performed on a 24 core custom configuration Thelio Mira workstation running Pop OS (System76, Denver, Colorado, USA). Each gantry angle required 2 days of computing using 20 cores. The calculations are scoring dose in air that has low density compared to condensed matter, and we want to resolve the scattered radiation. Both factors require a lot of source particles.

To simulate an axial CT scan, the gantry and its components were rotated to eight equal angles. Each angle used the maximum number of source photons in a thin beam inside the tube: 1 × 10^9^ photons. Therefore, for one complete rotation, the simulation is made with 8 × 10^9^ particles. The Eve positioning chair from Leo Cancer Care Cancer Care is not included in this simulation, but can easily be added later.

All the structures for the scanner and its supports are CAD (computer‐aided design) files, imported to the TOPAS parameters file as STL type files, summarized in Table 1. The CT scanner detectors are “Xtiles” (Detection Technology, Billerica, Massachusetts, USA) used for detection of the photons is a GOS scintillator with standard composition (atomic fractions) provided below in Table 1. The Steel is all assumed to be the Geant4 composition G4 Stainless Steel. The PCB (Printed Circuit Boards) boards were more challenging to model. A source was found for a good approximation of the chemical composition,^15^ and the density was assumed to be close to Al on average: 2.7 g/cc. Results were presented using MATLAB (version R2022b; Mathworks, Natick, Massachusetts, USA).

**TABLE 1:** This table lists the components of the Mary^®^ system used for the TOPAS simulation in this paper.

Component name	Component material	Color in Figure 2
Arm and Tilt Counter‐Weights	G4_STAINLESS‐STEEL	Green
Bearing	G4_STAINLESS‐STEEL	Gray
Bridge	G4_STAINLESS‐STEEL	Gray
Pillars	G4_STAINLESS‐STEEL	Gray
Platter	Aluminum	Gray
Shielding for Scatter	Tungsten	Green
Shielding Backstop	Tungsten	Green
Tube	Lead‐lined Aluminum	White
Tube Bracket	G4_STAINLESS‐STEEL	Gray
Yokes Left	G4_STAINLESS‐STEEL	Gray
Translation Structure	G4_STAINLESS‐STEEL	Gray
High Voltage Power Supply Bracket	G4_STAINLESS‐STEEL	Gray
Translation Counter Weights, dynamic stacks for Translating	Lead	Gray
Translation Counter‐Weights, dynamic plates for Translating	G4_STAINLESS‐STEEL	Gray
Collimator	Tungsten	Yellow
Collimator Subplate	G4_STAINLESS‐STEEL	Gray
Xtile (detector) Scintillator Layer	GOS [Gd(0.4), S(0.4), O(0.2)]	White
Antiscatter Grid	Tungsten	Yellow
Spine Plate	G4_STAINLESS‐STEEL	Green
Buncher Boards for the DAS	PCB [C(0.05), O(0.3), Al(0.4), Si(0.15), S(0.01), Cu(0.05), Sn(0.04)]	Orange
Driven Pully	G4_STAINLESS‐STEEL	Green
Scatter Phantom	water	Blue

Note that the dose is deposited assuming air everywhere, but the calculation involves the correct materials and geometries from the various components. Therefore, the isodose lines that result from this simulation only really have meaning where there is actually air at that location. Detectors and dosimeters that are calibrated for dose in air, exposure, or air kerma can directly use these calculations.

### 2.3 Experimental measurements

The calculation results, the isodose line values, are normalized to the survey meter value at 1 m away from scattering object, normal, out the bore. A RadEye G20‐ER10, Thermo Scientific survey meter was used. It is calibrated to provide exposure to air for these diagnostic x‐ray energies. The units are either in exposure rate (R/h) or equivalent dose rate (Sv/h) with assumptions, but fundamentally, the device is calibrated for an air kerma based measurement. Therefore, even the equivalent dose rate shown is really a calibrated air kerma measurement.

The value 1 m below the isocenter in the Mary scanner is equivalent to the same value in a typical recumbent scanner out of the bore along the couch. This 1 m out of the bore value is used in modern protocols as follows. The scatter from that phantom will ideally be nearly isotropic except for gantry attenuation. One can use the maximum of that value over any angle by measuring it 1 m at 45 or 90 degrees typically.

(1a) $$K_{s}^{1}=\;k\left(\frac{L}{p}\right)N\left(mAs\right)CTDI_{100}^{norm},$$

(1b) $$K_{s}=K_{s}^{1}\;{\left(\frac{1\left(m\right)}{d\left(m\right)}\right)}^{2}.$$

Here, *N* is the number of scans (patients) for a given time period, each with an average mAs of integrated tube current, used in conjunction with a normalized CTDI measurement. The quantity *L* is the scan length, relative to a CT pitch factor, *p*. The quantity *k* is the scatter fraction per length, a proportionality constant relating the line‐integrated absorbed dose in the phantom to the scatter air kerma measured at 1 m out of the CT bore. It is usually approximated with canonical values of 9E‐5 for the head phantom and 3E‐4 for the body phantom as recommended in the NCRP 147 report.^16^

Note that in the above protocol description, there is no need to use the gantry attenuation, no explicit angle to consider. One just uses the 1 m out value for most radiation protection protocols, and assumes a worst case of no attenuation from the gantry. In these protocols, the gantry is not explicitly considered, but manufacturers are expected to characterize this gantry attenuation.

### 2.4 Characterization of the gantry attenuation

Most manufacturers supply isodose lines for CT scanners are for a single fixed orientation of the gantry. Because the Mary scanner tilts, the authors propose that a maximum intensity set of isodose lines are best for calculating site planning needs for a movable gantry CT scanner such as the one Leo Cancer Care is making: the maximum intensity of dose in each voxel for all possible gantry angles. Since only ±15 degrees tilt is available now, a maximum dose intensity for ±15 degrees and 0 degree is used.

There were two ways that the gantry attenuation was characterized. First, the procedure laid out in Harpen (1998)^17^ provides for an analytical‐based calculation compared to measured isodose lines. His method uses a cleverly integrated Klein‐Nishina probability for the energies used for CT scanners. A single representative isodose line at one orientation is used to compare with other, more typical CT scanners in the Harpen (1998) treatment.

The other way to characterize the gantry and compare to other scanners is to plot the dose at a constant radius, say 1 m from isocenter. The path of the plot is a circle of radius equal to 1 m that goes around the gantry. However, in this case, a maximum intensity plot is less useful since other scanners to compare with will consider only a stationary gantry in the literature.

## 3 RESULTS

### 3.1 Experimental measurements

At the time of writing of this paper, we measured this 1 m dose rate for 140 kVp, measured below the isocenter, to be 0.04 microSv/mAs or 2.4 microSv/mA*min or 0.27 mR/mA*min. This value of 2.4 microSv/mA*min is just the quantity known as the scattered air kerma per patient at 1 m: *κ*_s_^1^. This value was used to normalize the whole dose scoring space. The value above the isocenter was a factor of 1.7 less than the value below isocenter.

### 3.2 Resulting scatter dose maps

**FIGURE 3:**
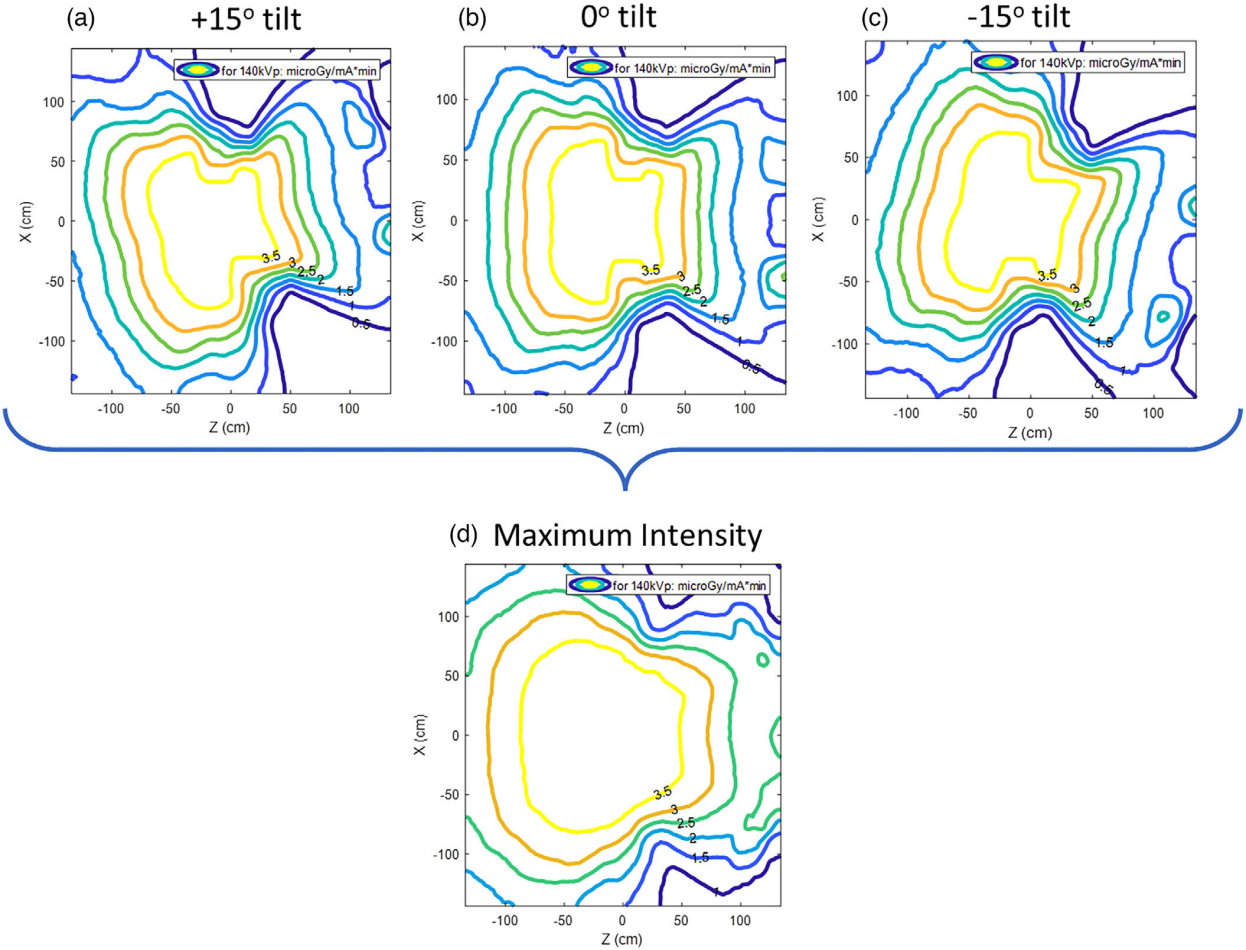
Isodose maximum intensity (d), and for separate tilt angles, air kerma central sagittal slices within the dose scoring parallel world of all air for all gantry angles and tilt angles of ±15 degrees (a and c) and 0 degrees (b).

**FIGURE 4:**
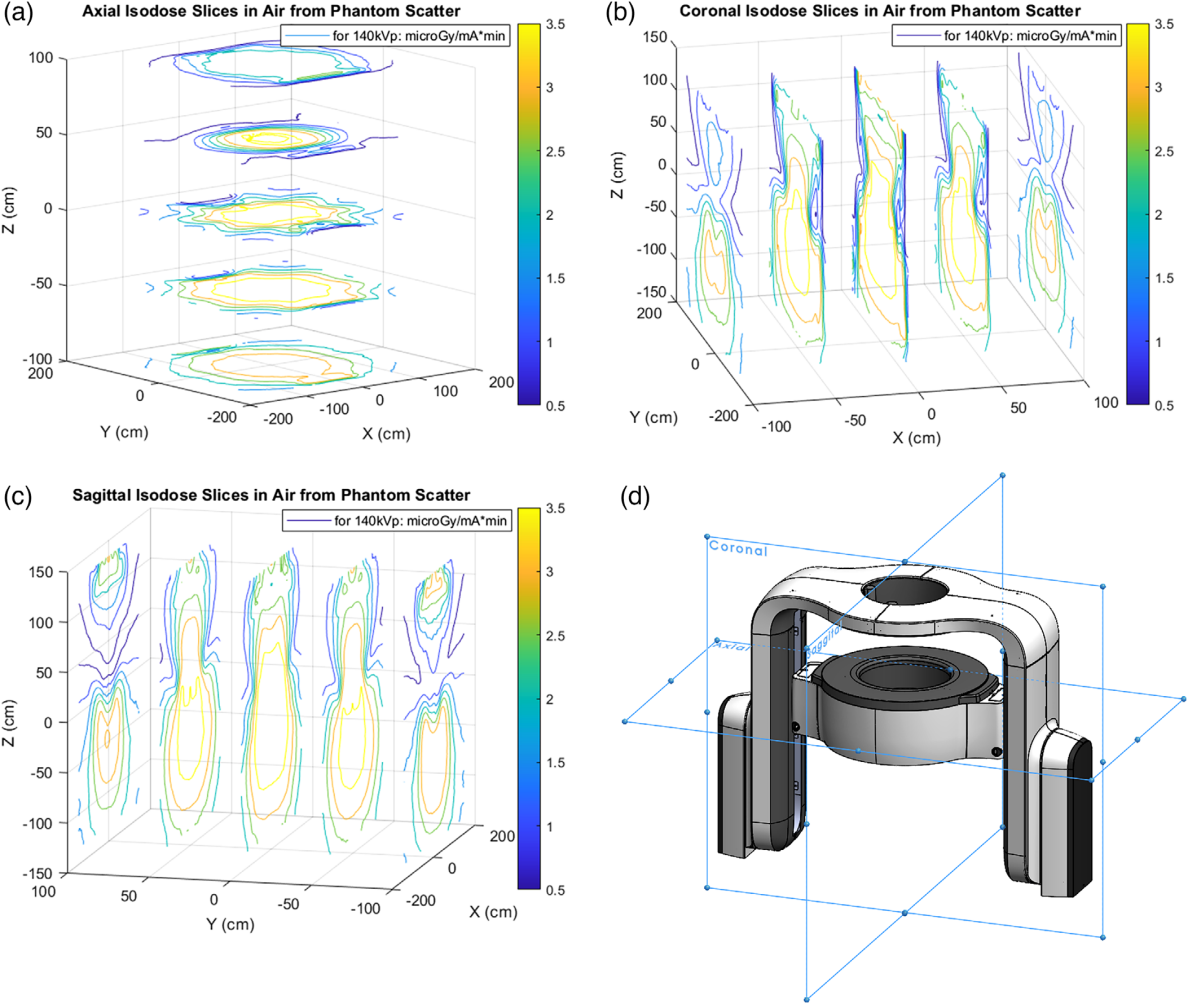
Isodose maximum intensity air kerma slices within the dose scoring parallel world of all air for all gantry angles and tilt angles of ±15 and 0 degrees. The energy fluence is all correct to the full calculation space, just scored only in air as shown: “a” are axial slices in height. Note the eight gantry locations are easy to see in the middle slice from the tube influence on the isodose lines; “b” coronal slices that include the arm supports that act to tighten the isodose lines on the sides; “c” are sagittal slices, and these are most similar to isodose lines from typical scanners that do not have arm supports. The middle slices in “a,” “b,” and “c” are shown in the picture of the upright scanner in the zero tilt position, but the isodoses are maximum intensity for all tilts in the initial product release. In “d,” the scanner is pictured for perspective.

Figure 3 shows the isodose lines in a central sagittal slice: ±15 and 0 degrees tilt, and the resulting slice of the maximum intensity image made from the other three. Note that the calculation is fully 3D: shown in Figure 4: the maximum intensity isodose lines in whole volume. The maximum intensity image represents a worst case, useful for shielding estimates. This study proposes this method as most appropriate for a moving gantry system like the Mary CT scanner from Leo Cancer Care.

### 3.3 Characterization of the gantry attenuation

**FIGURE 5:**
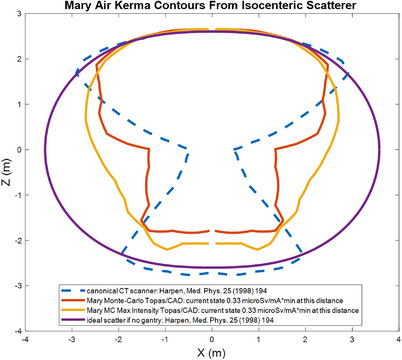
The isodose for the Leo Cancer Care Mary^®^ system shown in comparison to the figure in Harpen (1998). The maximum intensity isodose line here is for 0, and ±15 degrees.

Figure 5 shows the gantry affected isodose lines compared to those in Harpen (1998).^17^ The figure inset to his Figure 2 is reproduced here along with the Leo Cancer Care scanner for 0 degree tilt and the Maximum Intensity isodose for the three tilts: ±15 degrees and 0 degree for comparison, all to the ideal case of no gantry attenuation at all. The canonical CT scanner isodose lines used here are the same as in his paper and represent supplied isodose measurements from an anonymous manufacturer. The Leo Cancer Care scanner is tilted upright from the scanner used for those isodose lines, but our calculations do not include the influence of the floor, walls, and other items such as the upright chair positioning system at this time.

Note that the measurement ratio of 1 m of below the gantry reading to the 1 meter above the gantry above reading is 1.7. This value is similar to other scanners (see Figure 2 of Wallace et al., 2012^18^: most scanners shown have this ratio near 1.5), but not the same as the “canonical scanner” in Harpen (1998)^17^ that has this value as unity effectively.

**FIGURE 6:**
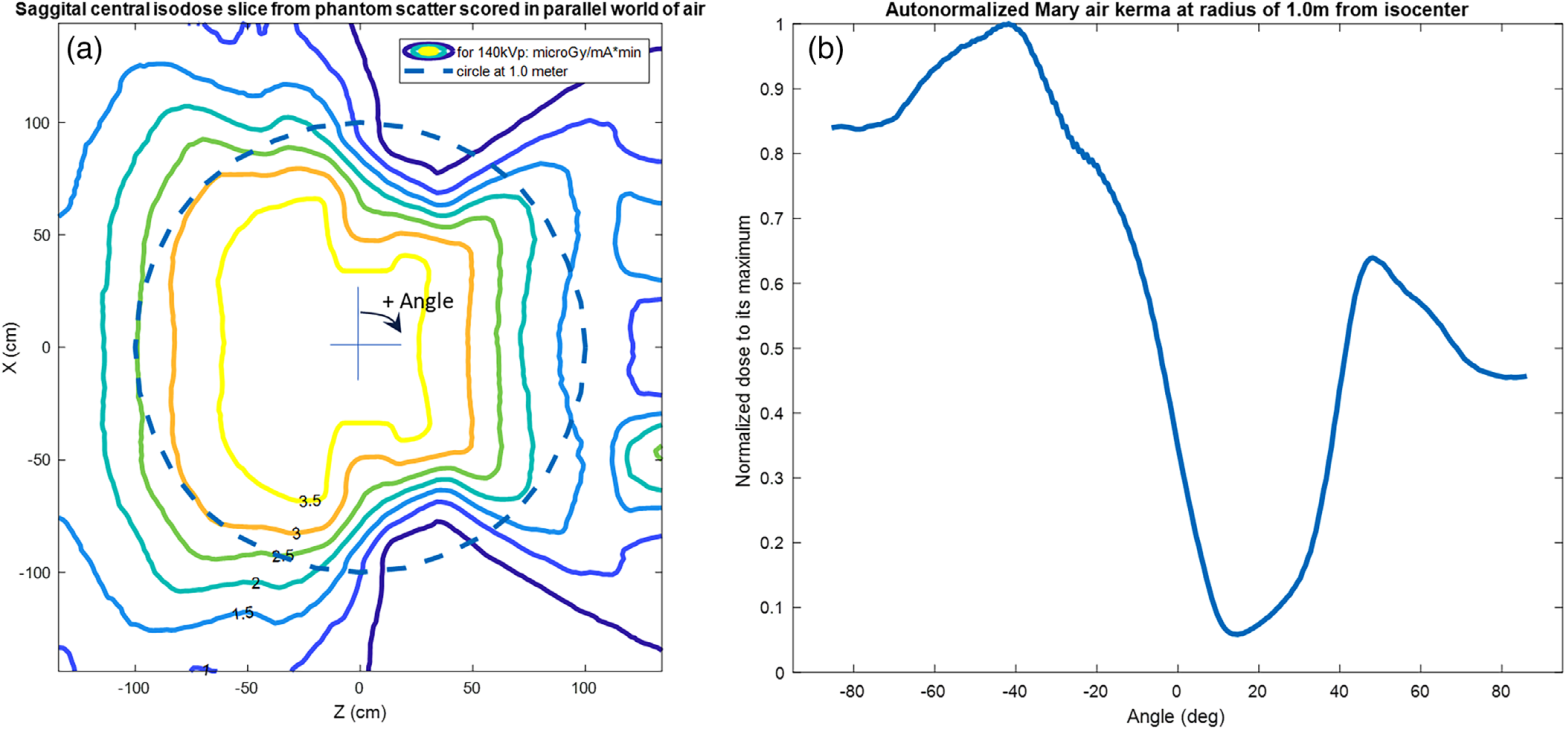
The modeled isodose lines from isocenter in a central sagittal slice for the Leo Cancer Care Mary^®^ system for a 0‐degree tilt angle (a). The overlaid circle indicates 1 m from the isocenter at all angles in this slice. In (b) are the values around the circle of 1 m radius distance from isocenter covers the gantry parts and so provides a calculation of gantry attenuation without the extra attenuation from the arms and supports. It is for the case shown in (a). Note that the dose is calculated with correct materials and geometry, but the dose is scored in a parallel world air box in TOPAS. The −90 degree position is straight down, and +90 degrees is straight up.

Figure 6 shows the value of the dose around the influence of the gantry at 1 m from isocenter all around the gantry. For up to 18 different typical CT scanners (Watanable et al., 2017,^19^ using Table 3), the average attenuation factor, corrected for distance, relative to the primary beam is a reduction of (6.8 ±3.5)%. The Leo Cancer Care upright scanner is about the same, see Figure 6b. The tube shielding itself is typically better than the gantry shielding by an order of magnitude and so is usually ignored compared to patient or phantom scatter. The Monte‐Carlo calculation though does include the tube leakage implicitly.

## 4 DISCUSSION

The spectrum used for the calculations in this paper was not perfect. The gantry was only populated with its largest or most significant objects, and the filtering was not completely developed. The values presented here are not intended to be precise or completely accurate to the final device. Even if the values were accurate and precise, there are other factors that contribute to the usefulness of the calculation here.

If one uses these isodose curves in Figure 4, it is possible to reduce the overall shielding thickness in some areas.^17^ However, few sites would do that. If one were to upgrade or change scanners, place a scanner in a new orientation, or purpose the room for something else entirely, the shielding would likely be sub‐optimal. The usual conservative approach is to put the maximum required shield thickness needed for one place, in all places. Note that Harpen (1998)^17^ does not specify the manufacturer for the “canonical” isodose curve shown, and it differs from measured curves in Wallace (2012), Figure 2.^18^

The calculations here also include the Mary scanner itself. The tertiary scatter from the walls, the ceiling, the floor, and from the positioning chair are all down in magnitude by about an order of magnitude and would be considered a correction for the purposes of radiation protection (BIR/IPEM 2000^20^), especially since the gantry attenuation is often ignored in modern protocols for radiation protection.

The NCRP report number 147^16^ suggests using a CTDI measurement (with or without a DLP calculation that uses CTDI volume instead of CTDI 100) to perform radiation protection calculations for the room for the photon scatter from a CTDI phantom (dimensions, etc.) placed at isocenter. Further, since head scans produce only a fraction of the scatter dose,^21^ many people only consider a body scan for radiation protection purposes, and so CTDI 100 is often all that is needed for radiation protection purposes. These phantoms are not anthropomorphic.

Also, the k values in Equation 1a are really scanner‐specific, actually implicitly including gantry leakage.^21^ The actual values can vary by tens of percents from the canonical value. The NCRP formalism often underestimates the dose and the shielding needed by up to tens of percents for other reasons too.^19^, ^21^ For radiation protection purposes, these uncertainties are not impactful. In fact, in the past, it was common to approximate the scatter air kerma at 1 m out of the CT bore with only one significant digit: Page 493 of Turner (2007).^22^

The method presented in this paper, when corrected for spectrum with final filtering and expanded with more gantry components and room boundaries, is capable of great accuracy and precision. Recently, artificial intelligence (AI) is used to help with the calculations for dose, denoising, and all the other outputs of these codes.^23^ Many other groups have used Monte Carlo for the design of a CT scanner and its environment. (e.g., ref. 24) The details of how the gantry attenuates and the methods presented here are also potentially useful to manufacturers in addition to customers on site.

## 5 CONCLUSION

For most users who need to plan for shielding, not much has to change from a standard scanner except for the general upright nature of the scanner. Moreover, the top‐heavy gantry and the bridge atop the scanner arms provide some extra shielding to help with scatter dose towards the ceiling.

## AUTHOR CONTRIBUTIONS

Authors Michael Kissick, Costanza Panaino, and Anthony Criscuolo all had creative roles in the TOPAS modeling. Michael Kissick performed the data analysis with Matlab. Authors John Hayes and Carson Hoffman helped with measurements at 1 m. Authors Rock Mackie and Niek Schreuder are senior authors that provided advice that help with the discussion and planning of the study.

## CONFLICT OF INTEREST STATEMENT

The authors declare no conflicts of interest.
